# Dexmedetomidine Protects against Ischemia and Reperfusion-Induced Kidney Injury in Rats

**DOI:** 10.1155/2020/2120971

**Published:** 2020-04-03

**Authors:** Naren Bao, Di Dai

**Affiliations:** ^1^Department of Anesthesiology, The First Hospital of China Medical University, 155 Nanjing North Street, Shenyang, China; ^2^Department of Laboratory Medicine, The First Hospital of China Medical University, 155 Nanjing North Street, Shenyang, China

## Abstract

Acute kidney injury (AKI), a clinical syndrome, is a sudden onset of kidney failure that severely affects the kidney tubules. One potential treatment is dexmedetomidine (DEX), a highly selective *α*_2_-adrenoreceptor agonist that is used as an anesthetic adjuvant. It also has anti-inflammatory, neuroprotective, and sympatholytic qualities. The aim of this study was to establish whether DEX also offers protection against ischemia and reperfusion- (I/R-) induced AKI in rats. An intraperitoneal injection of DEX (25 *μ*g/kg) was administered 30 min prior to the induction of I/R. The results indicate that in the I/R rats, DEX played a protective role by reducing the damage to the tubules and maintaining renal function. Furthermore, in response to I/R, the DEX treatment reduced the mRNA expression of TNF-*α*, IL-1*β*, IL-6, and MCP-1 in the kidney tissues and the serum levels of TNF-*α*, IL-1*β*, IL-6, and MCP-1. DEX also reduced the levels of oxidative stress and apoptosis in the tubular cells. These results indicate that in response to I/R kidney injury, DEX plays a protective role by inhibiting inflammation and tubular cell apoptosis, reducing the production of reactive oxygen species, and promoting renal function.

## 1. Introduction

Acute kidney injury (AKI) can be a consequence of major surgery. It significantly increases the risks for morbidity and mortality [1]. AKI can result in arrhythmia, cerebral edema, hyperkalemia, renal insufficiency, and water intoxication. All of these conditions pose a risk of death [2]. Few studies have reported on acute stress-induced AKI; thus, research into the underlying mechanisms and effective treatments is needed.

AKI can be initiated by ischemia and reperfusion (I/R). During I/R, the renal tubular epithelial cells experience disturbed cell polarity and cytoskeletal integrity, disrupted cell-cell and cell-matrix interactions, mitochondrial damage, and increased reactive oxygen species (ROS) synthesis [3–5]. Oxidative stress resulting from acute restraint stress has been determined to lead to hippocampal and hepatic damage [6]. Because oxidative stress can promote apoptosis, it also has the capacity to cause AKI. Indeed, a number of pathological kidney injuries have been attributed to apoptosis [6]. This finding has stimulated inquiries into the role of oxidative stress and apoptosis in the pathological process of I/R-induced kidney injury.

Dexmedetomidine (DEX) possesses several properties that are potentially beneficial for treating AKI. It is a potent and highly selective *α*_2_ adrenergic agonist with analgesic, sedative, and antisympatholytic characteristics [7, 8]. The distal and proximal tubules of the kidney, as well as the peritubular vasculature, are rich in *α*_2_-adrenoceptors [7, 8]. Using animal models, studies have explored the effects of DEX following I/R injury. As well as being beneficial for tubular architecture and function and tubular epithelial cell apoptosis, DEX reduces the synthesis of ROS and inhibits the secretion of proinflammatory cytokines [9]. Despite DEX's capacity to alleviate renal I/R injury following surgery, the mechanism by which it acts in AKI has yet to be elucidated.

The purpose of this study was to explore DEX's protective effects against renal I/R injury in rats. In addition, it is aimed at identifying the mechanisms by which DEX modulates apoptosis, inflammatory cytokines, and ROS.

## 2. Materials and Methods

### 2.1. Animals and Experimental Design

The study observed the China Medical University's guidelines for the use of laboratory animals. Approval was sought from and granted by the Ethics Committee on Animal Experiments of The First Hospital of China Medical University.

Eighteen healthy 7–8-week-old male Sprague-Dawley rats weighing 220–270 g were used. They were accommodated in a laboratory (22°C ± 1°C, 12–12 h light-dark cycle) in pathogen-free housing for one week prior to the experiment. Food and water were provided ad libitum until 12 h before the experiment. The food was then withdrawn; however, the water was available.

The rats were sedated and analogized with 10% chloral hydrate (0.3 ml/100 g) and 10 *μ*g/kg sufentanil citrate (Renfu Pharmaceutical, China, Lot 81A06031) injected intraperitoneally (i.p.). To improve perioperative analgesia, 0.2% ropivacaine (AstraZeneca AB, Sweden) was used for local infiltration before surgical incision and after suture. The rats' temperatures were measured, and they were sustained at 37°C (±1°C) with a heat pad. The heart rates were monitored with subcutaneous electrodes. Arterial pressure was invasively monitored by a 24 G trocar that was placed in the left femoral artery. The exclusion criteria were applied to the rats that had heart rates slower than 200 beats per min (bpm) for more than 5 min or mean blood pressures (MAP) less than 55 mmHg. To induce renal I/R injury, the right renal pedicle was clamped for 45 min, and the left was surgically removed.

After the removal of the arterial clip, the color of the remaining kidney was observed. A change from dark purple to reddish brown within 5 min signaled the successful restoration of blood perfusion. Each rat was administered a 0.5 ml saline i.p. injection every 2 h until it awoke or the specimen was collected. The animals were euthanized at 24 h after the I/R, and abdominal aortic blood samples were collected immediately. To remove the cellular elements, the blood samples were left on ice for 2 h and then centrifuged for 15 min (3,000 g, 4°C). The serum was stored at −80°C. The kidneys were harvested following transcardial perfusion with ice-cold heparinized saline. A part of the renal tissue sections were fixed in paraformaldehyde and embedded in paraffin wax; 4 *μ*m sections were removed and stained with hematoxylin and eosin. In addition, a terminal deoxynucleotidyl transferase deoxyuridine triphosphate nick-end labeling (TUNEL) assay was performed. The remainder of the renal tissue was maintained at −80°C until further analysis.

The rats were randomly assigned to one of three groups (*n* = 6 per group):

Group 1.Sham control (Sham): The rats received 0.5 ml saline i.p. injections prior to sham surgery. The renal vessels were not clamped.

Group 2.Renal I/R group (I/R): The surgery was performed as previously described.

Group 3.DEX+I/R group (DEX+I/R): At 30 min prior to the initiation of renal ischemia, 25 *μ*g/kg of DEX was administered by i.p. injection.

### 2.2. Renal Histology

The kidneys were postfixed with 10% buffered formalin, dehydrated in graded ethanol solutions, embedded in paraffin, sectioned, and stained with hematoxylin and eosin. Acute tubular necrosis was graded on a 0 to 4 scale on the basis of the damage to the cortex or outer medulla: 0 = normal, 1 = minimal damage (<5% involvement), 2 = mild damage (5–25% involvement), 3 = moderate damage (25–75% involvement), and 4 = severe damage (>75% involvement) [10].

### 2.3. Renal Function

Serum creatinine (CREA) and serum urea nitrogen were quantified with a UniCel DxC800 Synchron device (Beckman, USA). Standard enzyme immunoassay kits (BioPorto Diagnostics, Gentofte, Denmark) were used to establish the serum concentrations of the neutrophil gelatinase-associated lipocalin (NGAL) and cystatin C.

### 2.4. Reverse Transcription Polymerase Chain Reaction

Reverse transcription polymerase chain reaction (RT-PCR) was used to determine the expression levels of TNF-*α*, IL-1*β*, IL-6, and MCP-1 in the kidneys. The total cellular RNA was extracted with TRIzol (Invitrogen), and the cDNA was synthesized with a Moloney murine leukemia virus (M-MLV) reverse transcription kit (Promega). Quantitative real-time PCR was performed through the use of an IQ SYBR Green Supermix reagent (Bio-Rad, USA) with a Bio-Rad real-time PCR machine. The manufacturer's instructions were followed. The data were analyzed by the use of the −*ΔΔ*CT method. Glyceraldehyde 3-phosphate dehydrogenase (GAPDH) was used as a housekeeping gene against which the expression levels of the target genes were normalized.  Table 1 presents the primer sequences.

### 2.5. Kidney Oxidative Stress Analysis

A homogenate of the kidney tissue was made to measure the concentration of glutathione (GSH) and malondialdehyde (MDA). The homogenate was also used to identify superoxide dismutase (SOD) antioxidant enzyme activity. The manufacturer's instructions for the corresponding assay kit (Nanjing Jiancheng Bioengineering Institute, Nanjing, China) were followed.

### 2.6. Serum Inflammatory Cytokine Analysis

The serum inflammatory cytokines were measured through the use of a commercial enzyme-linked immunosorbent assay (ELISA) kit from Nanjing Jiancheng Bioinstitute (Nanjing, Jiangsu, China) and a microplate reader at 450 nm (Thermo Scientific, Waltham, MA). The manufacturer's guidelines were followed.

### 2.7. Terminal Deoxynucleotidyl Transferase Deoxyuridine Triphosphate Nick-End Labeling (TUNEL) Assay

To detect the apoptotic tubular epithelial cells, an in situ TUNEL assay was used in accordance with the manufacturer's instructions (In Situ Cell Death Detection Kit, POD, Roche, Switzerland). The sections that had been fixed in paraffin were dewaxed. They were then rehydrated with xylene and a graded series of ethanol and double-distilled water. The sections were treated with proteinase K (20 *μ*g/ml) at room temperature for 15 min before being rinsed twice with phosphate buffer saline (PBS). After the slide was dried, 50 *μ*l of TUNEL reaction mixture was applied. The slides were incubated at 37°C in a humidified chamber for 60 min and then rinsed three times with PBS. Converter-POD (50 *μ*l) was added to the specimen, and a coverslip was applied before incubation at 37°C in a humidified chamber for 30 min. The samples were again rinsed three times with PBS and then stained with a diaminobenzidine (DAB) substrate (Zhongshan, Beijing, China). After 10 min, the DAB was removed by being rinsed three times with PBS. Hematoxylin or methyl green stains were applied and then rinsed with tap water almost immediately. ImageJ software (version 1.38; National Institutes of Health, Rockville, MD) was used for counting the number of TUNEL-positive cells in an objective grid in regions of 10 randomly selected sections. A 40x objective lens was used for the counts, which were performed by a researcher who was blinded to the experiment aims. The data from the average of these 10 counts were analyzed.

### 2.8. Statistical Analysis

The data were analyzed in IBM SPSS Statistics for Windows, version 22.0 (SPSS Inc., Chicago, IL, USA). The results are presented as means and standard deviation (SD). One-way analysis of variance (ANOVA) was used to compare multiple sets of data. Statistical significance was set at *p* < 0.05. The graphs were created in GraphPad Prism 7.00 (GraphPad Inc., San Diego, CA, USA).

## 3. Results

**Figure 1:**
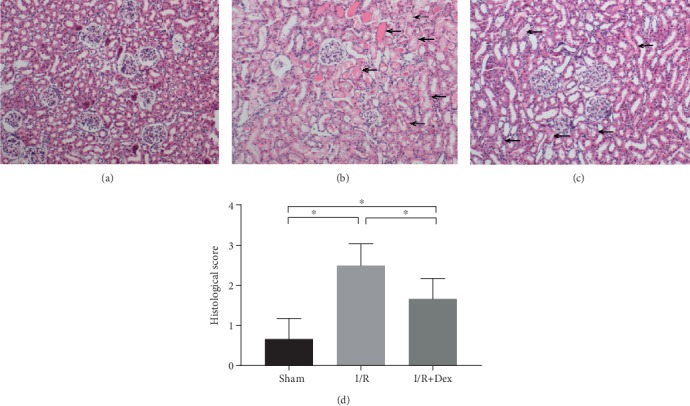
Dexmedetomidine (DEX) preconditioning showed renoprotective effects histologically (10x). Renal injury was inflicted in the experimental group by removing the left kidney and clamping the right renal artery for 45 min (renal ischemia-reperfusion (I/R)). In the sham control group, both renal pedicles were dissected without occlusion. DEX was administered 30 min prior to ischemia. Representative microphotographs were taken from (a) the sham control group, (b) the I/R group, (c) the DEX (25 *μ*g/kg)+I/R group, and (d) the quantification of the histological scores following I/R in rats. The histological damages were indicated by black arrows. The data are the mean ± SD (*n* = 6).∗*p* < 0.05.

To confirm the effects of DEX on I/R-induced renal injury, 25 *μ*g/kg of DEX was administered by i.p. injection 30 min before I/R.  Figure 1 presents the histological images through which kidney injury was determined. The evidence provided by the hematoxylin and eosin (H&E) stains indicates that the histological injuries to the I/R rats' kidneys included focal renal hemorrhage, focal tubular necrosis, neutrophil infiltration, and vacuolar degeneration of the renal tubular epithelial cells (*p* < 0.05). The histological damage in the DEX-treated rats was lesser (*p* < 0.05) than that in the I/R rats.

**Figure 2:**
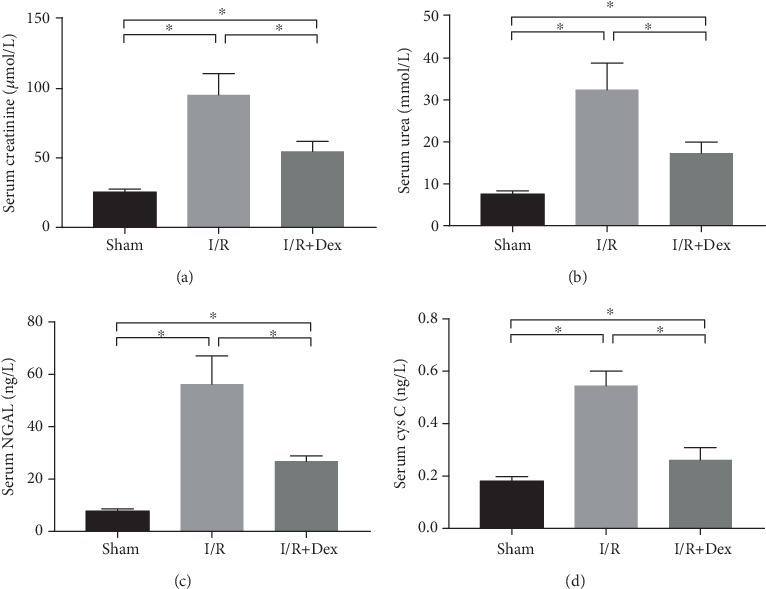
Renal function in rats subjected to the sham procedure, untreated ischemia-reperfusion (I/R), or I/R with dexmedetomidine (DEX) treatment. Levels of (a) serum creatinine, (b) serum urea nitrogen, (c) serum neutrophil gelatinase-associated lipocalin (NGAL), and (d) cystatin C (Cys C) in the sham control, I/R, and DEX (25 *μ*g/kg)+I/R groups. The data are the mean ± SD (*n* = 6). ∗*p* < 0.05.

As is shown in Figure 2, the marked increase in serum CREA (*p* < 0.05) and urea nitrogen (*p* < 0.05) was indicative of I/R-induced renal dysfunction. However, these serum markers were significantly lower (*p* < 0.05) in the DEX-treated rats than in the I/R rats. This suggests that the renal function in the DEX-treated rats was maintained to some extent after I/R induction. Furthermore, the serum NGAL and cystatin C levels in the I/R-induced rats were significantly higher (*p* < 0.05) than those in the sham group. However, they were significantly lower (*p* < 0.05) in the DEX-treated rats than in the I/R-induced rats (Figure 2).

**Figure 3:**
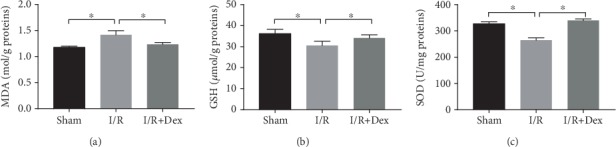
Extent of oxidative stress in rats subjected to the sham procedure, untreated ischemia-reperfusion (I/R), or I/R with dexmedetomidine (DEX) treatment. Levels of (a) malondialdehyde (MDA), (b) glutathione (GSH), and (c) superoxide dismutase (SOD) in the sham control, I/R, and DEX (25 *μ*g/kg)+I/R groups. The data are the mean ± SD (*n* = 6). ∗*p* < 0.05.

The effects of DEX on oxidative stress were also evaluated. As is illustrated in Figure 3, the MDA concentrations in the I/R-induced rats were significantly higher (*p* < 0.05) than those in the sham group. However, in the I/R rats treated with DEX, the levels of oxidative stress were lower (*p* < 0.05) than those in the I/R rats. The GSH and SOD activity levels in the I/R group were significantly lower (*p* < 0.05) than those in the sham controls. Indeed, the GSH concentrations and SOD activity levels in the I/R group treated with DEX were significantly higher (*p* < 0.05) than those in the I/R group.

**Figure 4:**
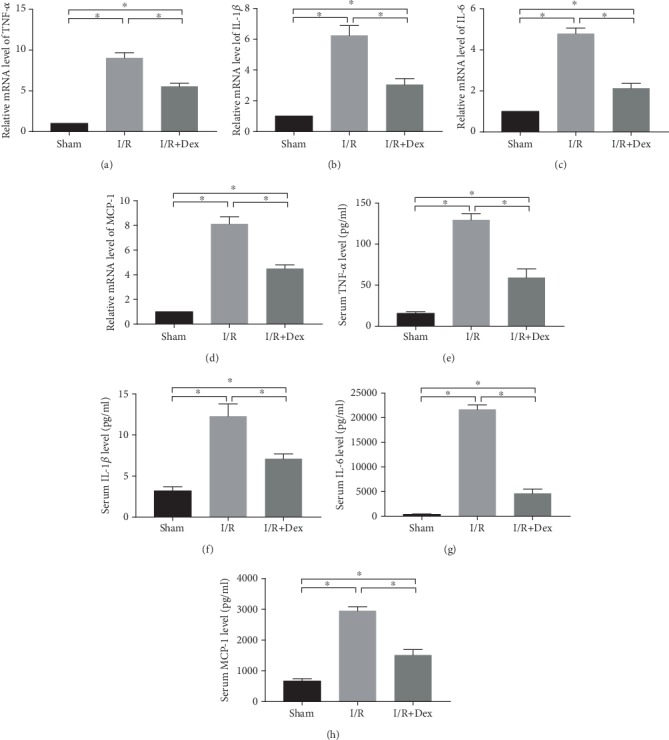
The mRNA levels of the inflammatory mediators in the rat kidneys or the serum levels of the inflammatory mediators subjected to the sham procedure, ischemia-reperfusion (I/R), or I/R with dexmedetomidine (DEX) treatment. Reverse transcription polymerase chain reaction (RT-PCR) tests of the mRNA levels of (a) IL-1*β*, (b) IL-6, (c) MCP-1, and (d) TNF-*α* in the kidney tissues from the sham control, I/R, and DEX (25 *μ*g/kg)+I/R groups. Enzyme-linked immunosorbent assay (ELISA) of the levels of (e) IL-1*β*, (f) IL-6, (g) MCP-1, and (h) TNF-*α* in the sera from the sham control, I/R, and DEX (25 *μ*g/kg)+I/R groups. The data are the mean ± SD (*n* = 6). ∗*p* < 0.05.

The effects of DEX on the expression of proinflammatory cytokines were evaluated. The RT-PCR results for the kidney samples indicated that the expression levels of IL-1*β*, IL-6, MCP-1, and TNF-*α* mRNA were significantly higher (*p* < 0.05) in the I/R-induced rats than in the controls (Figure 4). However, DEX exerted a modulatory effect. The mRNA expression of IL-1*β*, IL-6, MCP-1, and TNF-*α* (*p* < 0.05) in the kidney tissues of the rats that received DEX treatment was lower than that in the I/R-induced rats. The serum levels of IL-1*β*, IL-6, MCP-1, and TNF-*α* in the I/R rats were considerably higher than those in the DEX-treated I/R rats (*p* < 0.05). The serum levels of IL-1*β*, IL-6, MCP-1, and TNF-*α* (*p* < 0.05) in the rats that received DEX treatment were lower than those in the I/R-induced rats. This suggests that in the case of I/R injury, DEX exerts an inhibitory effect on the expression of inflammatory cytokines.

**Figure 5:**
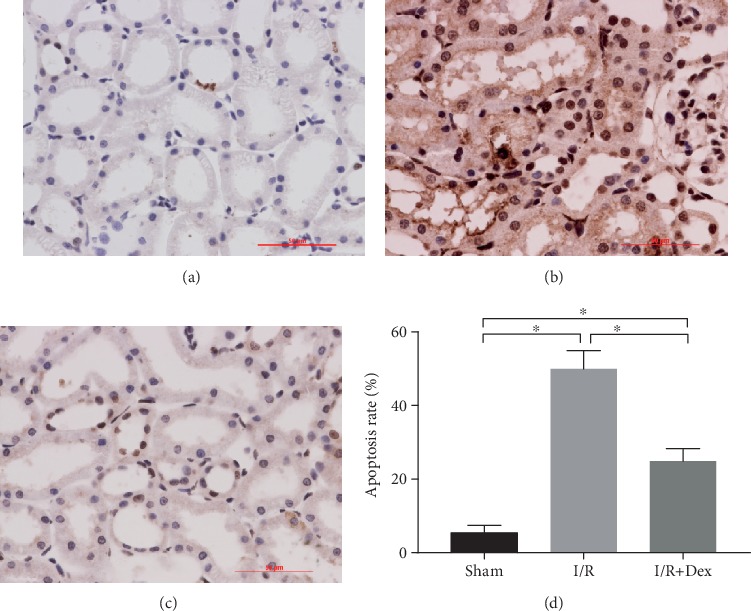
Apoptosis was detected in the rat kidneys via terminal deoxynucleotidyl transferase deoxyuridine triphosphate nick-end labeling (TUNEL) staining (40x) in rats subjected to the sham procedure, untreated ischemia-reperfusion (I/R), or I/R with dexmedetomidine (DEX) treatment. Representative microphotographs were taken from (a) the sham control group, (b) the I/R group, (c) the DEX (25 *μ*g/kg)+I/R group, and (d) the apoptosis rate following I/R in the rats. The data are the mean ± SD (*n* = 6). ∗*p* < 0.05.

The available evidence suggests that the pathogenesis of I/R injury is aggravated by the apoptosis of the tubular cells. Thus, the effect of DEX on tubular epithelial cell apoptosis in the I/R-induced rats was explored. The results of the TUNEL assay revealed that I/R injury was associated with a significant increase (*p* < 0.05) in the level of apoptosis in these epithelial cells. In contrast, there were fewer apoptotic cells in the kidney samples from the DEX-treated rats (*p* < 0.05) than in those from the I/R group (Figure 5).

## 4. Discussion

A sequela of cardiovascular and transplant surgery or shock is renal I/R injury that causes AKI. This results in longer hospital stays and even death [11]. The present study has demonstrated that pretreatment with DEX helps to maintain renal morphology and function in cases of I/R injury. When kidney I/R occurred, the levels of inflammatory mediators circulating in the blood were reduced, and the kidneys experienced less oxidative stress as a consequence of the administration of DEX. These findings suggest that DEX might be an appropriate pharmaceutical intervention to reduce acute injury-induced kidney damage.

Among the qualities of DEX are its anesthetic-sparing effect and facilitation of hemodynamic stability. Consequently, DEX is frequently administered as a sedative in perioperative and intensive care medicine. It is also applied as an anesthetic adjuvant [12]. DEX has wide clinical applications because its analgesic, hemodynamic, sedative, and sympatholytic qualities make it particularly useful for perioperative patients.

Other features are its anti-inflammation, antioxidant, and antiapoptotic effects on the brain, heart, lungs, and kidneys [13, 14]. The results of several animal model studies indicate that DEX also offers protection against I/R injury to the kidney [12, 15]. Other studies have highlighted the beneficial role of DEX in preventing renal injury in patients undergoing cardiovascular and other major surgical procedures [16]. These findings supplement those of earlier studies that reported significant increases in the CREA and BUN levels following I/R injury. This suggests impaired renal function. An examination of the histopathological evidence indicates that I/R causes kidney injury; however, DEX pretreatment can be mitigative.

A surfeit of oxygen-free radicals is produced by organisms in a state of stress. This disrupts the delicate balance between the oxidation and antioxidant systems [17]. The results of this study indicate that the MDA levels, an important biomarker of oxidative damage, are reduced and the GSH and SOD levels are increased by DEX. MDA is a useful indirect indicator of the extent of free radical-induced damage [18]. In contrast, GSH and SOD are important antioxidants [19]. The results suggest that I/R diminished the antioxidant defense system by increasing the MDA levels and reducing the GSH and GSH enzyme activity. This suggests that I/R could induce oxidative stress, which might be significant in the pathogenesis of AKI. The DEX treatment helped to protect against oxidative stress suggesting that it has antioxidative effects [20]. Therefore, DEX could provide protection against I/R-induced AKI.

Another manifestation of I/R injury is a heightened inflammatory response [21, 22]. This response aggravates the conditions because the macrophages, T cells, and other inflammatory mediators are recruited to the tissues injured by I/R [21, 22]. As the results of the tissue samples obtained from the kidneys of I/R-injured rats in this study have demonstrated, the expression of IL-1*β*, IL-6, MCP-1, and TNF-*α* mRNA was elevated. In contrast, the renal tissue samples from the rats that had received DEX following I/R exhibited lower IL-1*β*, IL-6, MCP-1, and TNF-*α* mRNA expression. Relationships among renal morphology, the serum levels of inflammatory mediators, and the state of cell apoptosis were found. The pathogenesis and progression of I/R injury are influenced by the proinflammatory cytokines, including IL-1*β*, IL-6, MCP-1, and TNF-*α* [23, 24]. DEX not only acts locally on the kidney's *α*_2_-adrenoceptors but also influences the anti-inflammatory reactions, thereby mitigating the damaging effects of I/R on the kidney.

Apoptosis, also known as programmed cell death, is a critical mechanism for maintaining cell stability; however, too much can damage the body [25]. Usually, apoptosis, an early event in kidney I/R injury, interacts with the subsequent inflammation and kidney injury [26]. In the present study, a significantly higher number of TUNEL-positive cells were retrieved from the rats with the I/R-injured kidneys than from those that received the DEX intervention. Studies have found that TNF-*α* causes injury to the kidneys [27, 28]. DEX might protect against AKI following I/R by stimulating an antiapoptotic effect.

The data obtained from this study demonstrate that DEX exerts a protective effect against I/R injury in rats by inhibiting tubular cell apoptosis and inflammation, lowering ROS production, and promoting renal function. These results suggest that DEX may play a role in the treatment of I/R-initiated AKI. However, DEX is not without side effects; through parasympathetic activation, it can contribute to bradycardia. How DEX can be best exploited to protect vital organs has yet to be established. Further research into its applications is justified.

## Figures and Tables

**Table 1:** Primers used in this study.

Primers	Sequences
TNF-*α*	Forward, 5′-CCC GGA ATG TCG ATG CCT GAGTG-3′
	Reverse, 5′-CGC CCC GGC CTT CCA AAT AAAT-3′

IL-1*β*	Forward, 5′-GCC CAT CCT CTG TGA CTC AT-3′
	Reverse, 5′-AGG CCA CAG GTA TTT TGT CG-3′

IL-6	Forward, 5′-TCT CGA GCC CAC CAG GAA CGA-3′
	Reverse, 5′-AGG GAA GGC AGT GGC TGT CA-3′

MCP-1	Forward, 5′-AGC ATC CAC GTG CTG TCT C-3′
	Reverse, 5′-GAT CAT CTT GCC AGT GAA TGAG-3′

GAPDH	Forward, 5′-AGG TCG GTG TGA ACG GAT TTG-3′
	Reverse, 5′-TGT AGA CCA TGT AGT TGA GGTCA-3′

## Data Availability

The data used to support the findings of this study are available from the corresponding author upon request.
